# Leflunomide plus low-dose prednisone in patients with progressive IgA nephropathy: a multicenter, prospective, randomized, open-labeled, and controlled trial

**DOI:** 10.1080/0886022X.2021.1963775

**Published:** 2021-08-15

**Authors:** Zhaohui Ni, Zhen Zhang, Zanzhe Yu, Fuming Lu, Changlin Mei, Xiaoqiang Ding, Weijie Yuan, Wei Zhang, Gengru Jiang, Min Sun, Liqun He, Yueyi Deng, Huihua Pang, Jiaqi Qian

**Affiliations:** a Department of Nephrology, Ren Ji Hospital, School of Medicine, Shanghai Jiao Tong University, Shanghai, China; b Department of Nephrology, Huashan Hospital, Fudan University, Shanghai, China; c Department of Nephrology, Shanghai Changzheng Hospital, Second Military Medical University, Shanghai, China; d Department of Nephrology, Zhongshan Hospital, Fudan University, Shanghai, China; e Department of Nephrology, Shanghai General Hospital, Shanghai Jiao Tong University, Shanghai, China; f Department of Nephrology, Shanghai Ninth People’s Hospital, School of Medicine, Shanghai Jiao Tong University, Shanghai, China; g Department of Nephrology, Xinhua Hospital, School of Medicine, Shanghai Jiao Tong University, Shanghai, China; h Department of Nephrology, Huadong Hospital, Fudan University, Shanghai, China; i Department of Nephrology, Shuguang Hospital Affiliated to Shanghai University of Traditional Chinese Medicine, Shanghai, China; j Department of Nephrology, Longhua Hospital, Shanghai University of Traditional Chinese Medicine, Shanghai, China

**Keywords:** IgA nephropathy, leflunomide, glucocorticoids, disease progression, proteinuria

## Abstract

**Background:**

Immunoglobulin A nephropathy (IgAN) is the most common cause of glomerulonephritis worldwide, and the optimal approach to its treatment remains a significant challenge.

**Methods:**

We did a prospective, randomized, open-labeled, multicenter, controlled trial, comprised of 3-month run-in, 12-month treatment, and 12-month follow-up phases. After 3-month run-in phase, patients with biopsy-confirmed IgAN at risk of progression were randomly allocated to LEF plus low-dose prednisone (LEF + prednisone group) or conventionally accepted-dose prednisone [prednisone(alone) group] Our primary outcome was 24-h urine protein excretion (UPE) and secondary outcomes were serum albumin (sALB), serum creatinine (Scr), and eGFR. Safety was evaluated in all patients who received the trial medications.

**Results:**

One hundred and eight patients [59 in LEF + prednisone group, 49 in prednisone (alone) group]were enrolled and finished their treatment and follow-up periods. There is no significant difference in the baseline level between the two groups. Compared with baseline, both groups showed a significant decrease in 24-h UPE (*p* < 0.01) and increase in sALB (*p* < 0.01), with stable Scr and eGFR throughout the 12-month treatment period. What’s more, these effects were sustained through the 12-month follow-up period. However, there was no difference in 24-h UPE, sALB, Scr, and eGFR between the two groups (*p* > 0.05). At 12 months, a difference in overall response rate, relapsing rate, and incidence of adverse events between the two groups was not significant.

**Conclusions:**

The efficacy and safety of LEF plus low-dose prednisone and conventionally accepted-dose prednisone in the treatment of progressive IgAN are comparable.

## Background

IgA nephropathy (IgAN) is the most common type of primary glomerular disease [1], accounting for about 40% of primary glomerular diseases in our country [2]. Patients with IgAN have a variety of clinical presentations, ranging from isolated hematuria to rapidly progressive kidney failure. Evidence shows that nearly 50% of IgAN is progressive and eventually develops into end-stage kidney failure (ESKD) in 10–20 years [3,4]. Furthermore, persistent proteinuria, hypertension, and reduced estimated glomerular filtration rate (eGFR) are major risk factors for IgAN progression to ESKD [5]. Barbour et al. considered patients with persist protein excretion >1 g/d after optimization of conservative measures, including blood pressure control and inhibition of the renin-angiotensin system, worse renal function, and histological lesions to be at significantly increased risk of disease progression in IgAN [6]. Thus, patients with progressive IgAN should be treated aggressively.

Kidney Disease: Improving Global Outcomes (KDIGO) guidelines recommend angiotensin-converting enzyme inhibitors (ACEIs) or angiotensin receptor blockers (ARBs) as first-line treatment for patients with IgAN with proteinuria of more than 1 g/day (recommendation level 1B) [7]. For patients with overt proteinuria of more than 1 g/day and eGFR > 50 mL/min/1·73 m^2^, 6 months’ treatment with high-dose systemic glucocorticoids is recommended (recommendation level 2 C) [7]. However, high-dose systemic glucocorticoids are associated with an increase of adverse events, such as serious infections, hypertension, osteoporosis, weight gain, and diabetes [8]. Accordingly, the Therapeutic Evaluation of Steriods in IgA Nephropathy Global (TESTING) trial was forced to stop early after recruitment, with a significantly higher risk of serious, fatal adverse events in the high-dose corticosteroid group [9]. Thus, the chosen dose of glucocorticoids for the treatment of IgAN is extremely important.

IgAN is recognized as an autoimmune kidney, which is one of the rationales for the use of immunosuppression in IgAN treatment [10]. The evidence for immunosuppressive therapy in IgAN is still insufficient. The benefit of systemic immunosuppression in treating IgAN has been questioned in the STOP-IgAN trial, which showed no significant effect of using immunosuppression both in terms of change in eGFR after 3 years of follow-up or the development of ESKD in patients with IgAN and persistent proteinuria with protein excretion 0.75 g/day, despite supportive care including blockers of the renin-angiotensin system (RAS). Recently, fewer studies were conducted to comprehensively assess the efficacy and safety of immunosuppressive therapy in IgAN. Hou et al. indicated that mycophenolate mofetil plus prednisone could reduce adverse events in patients with IgAN, and the histopathologic lesions were taken into consideration in the study [11,12]. However, clinical and laboratory indicators, such as 24-h urine protein excretion (UPE), eGFR, serum albumin (sALB), serum creatinine (Scr), were not taken into consideration in this study, and as such, it is impossible to determine whether its conclusions apply to patients with overt proteinuria and reduced eGFR. Moreover, our previous single-center study showed that leflunomide (LEF) combined with low-dose corticosteroid could reduce proteinuria and severe adverse events during long-term follow-up [13]. Thus, a large-scale, multi-center randomized controlled trial (RCT) is needed to further evaluate the efficacy and safety of LEF for the treatment of patients with IgAN who are at risk of progression to ESKD.

LEF is a new immunosuppressant and anti-inflammatory drug, by blocking the *de novo* synthesis of pyrimidines which interfere with DNA synthesis; and inhibiting inflammatory cytokine-mediated activation of Nuclear Factor κB and protein tyrosine phosphorylation. It is now widely applied in rheumatoid arthritis, systemic lupus erythematosus, and organ transplant rejection, and achieves approval effects. However, the efficacy and safety of LEF in the treatment of progressive IgAN are still unclear, and large-scale, multi-center randomized controlled study of progressive IgAN is limited [14,15].

Hence, we hypothesize that LEF might be effective for IgAN. Here, a prospective, multicenter, open, randomized, parallel controlled study was conducted to observe the clinical efficacy and safety of LEF plus low-dose prednisone in the treatment of progressive IgAN.

## Methods

### Patients

Patients were recruited from 12 renal units in Shanghai, China. They were aged 18–65 with biopsy-confirmed primary IgAN in recent 3 months, and with any one of the following indications for progression in IgAN: 24-h UPE > 1.0 g/day; eGFR < 60 mL/min per 1.73 m^2^ (calculated by CKD-EPI equation); and renal histological lesions defined as Lee’s IV, or glomerulus and/or segmental sclerosis ≥40%. Patients with any one of the following conditions were excluded: (a) rapidly progressive IgAN (IgAN with rapid renal function loss, characterized histopathologically by necrotizing capillaritis or active crescent formation >50%); (b) secondary IgAN, such as Henoch-Schonlein purpura nephritis, hepatitis-associated glomerulonephritis, and lupus nephritis, diabetic nephropathy, etc.; (c) receiving immunosuppressive and cytotoxic drugs for over 1 week or corticosteroid more than 20 mg/day for more than 4 weeks within 6 months; (d) eGFR <30 mL/min per 1.73 m^2^; (e) malignancy, HIV infection, acute central nervous system diseases, serious gastrointestinal diseases; (f) pregnancy or lactation. This study was approved by local ethics committees (reference number: [2004]12A) and all patients provided written informed consent before enrollment. The trial is registered at isrctn.org with the ISRCTN97636235 on 28 July 2006.

### Procedures

Before randomly allocation, eligible patients were enrolled into a 3-month run-in phase, during which, All patients received RAS blockade and RAS blockade was optimized by adjusting ACEIs and ARBs to a maximum recommended dose or maximum tolerated dose (in keeping with established clinical practice), to a target blood pressure of <130/80 mmHg. At the end of run-in, patients according to the inclusion criteria were randomly allocated to LEF plus low-dose prednisone (LEF + prednisone group) or conventionally accepted-dose prednisone group [prednisone (alone) group]. All patients continued optimized ACEIs or ARBs treatment throughout the trial. Patients in LEF + prednisone group received LEF 40 mg/day for 3 days, after which the dose was reduced to 20 mg/day and administered for 12 months, combined with oral prednisone 0.5–0.8 mg/kg/day (determined by the age and general condition of the patient) for 8–12 weeks with a maximum daily dose of 40 mg. Then prednisone was tapered by 5 mg, 2.5 mg by month to a maintenance dose of 10 mg per day. Patients in the prednisone (alone) group received oral prednisone 1 mg/kg/day for 8–12 weeks, which was tapered by 5 mg, 2.5 mg by month to a maintenance dose of 10 mg per day. The maximum daily dose of prednisone was 60 mg. The followed-up is 12 months. During the treatment, when the disease relapsed, it is allowed to maintain the prednisone unchanged for 4 weeks, or increase to the dose before relapsing for 2–4 weeks and, if necessary, temporary methylprednisolone was allowed (<1 g). During the follow-up period, the patients retreated with the original regimen when the disease relapsed.

### Allocation

Patients will be randomly assigned to either the LEF + prednisone group or the prednisone(alone) group at a 1:1 allocation ratio, using a computer-generated randomization schedule of permuted blocks of random sizes ranging from 4 to 10. The creation of the randomization sequentially numbered will be performed by persons not else involved in the trial. The final enrollment and subsequent allocation of participants will be conducted by investigators not taking part in any outcome assessment, who will be blinded to the randomization sequence at all times during the intervention period. Outcome assessors will not take part in any of the processes related to allocation.

### Outcome

Patients were randomized to the LEF + prednisone group or prednisone (alone) group using a computer algorithm method of permuted blocks. Demographics and baseline characteristics were collected at month 0. When recording clinical and laboratory characteristics at month 3, month 6, month 9, month12, month 24, the medications and adverse events were recorded at the same time. Standardized questionnaires at each visit were used to ask patients about the presence of specific LEF-related and corticosteroid-related adverse events.

The primary outcome was 24-h UPE and secondary outcomes were sALB, Scr, and eGFR.

Complete remission (CR) was defined as 24-h UPE < 0.3 g/d, with stable Scr (changes in Scr ≤15% of baseline values) and sALB ≥35 g/L; partial remission (PR) was defined as 24-h UPE decreased by 50% of the baseline value and ≥0.3 g/d, with stable Scr and sALB ≥30 g/L; No response (NR) was defined as a 24-h UPE > 3.5 g/d, or <50% reduction in baseline value, or Scr doubled. Relapse was defined as the reappearance of overt proteinuria, defined as >1.0 g/d or an increase of >50% from the lowest level of proteinuria after remission [16,17].

### Statistical analysis

Normal distribution variables were expressed by means ± *SD* and compared by *t*-test or ANOVA. Non-parametric variables were represented as median with range, and either the Mann-Whitney U test or the Kruskal-Wallis test was used. The chi-square test was employed for the categorical variables. Statistical analyses were performed using SPSS 13.0, with *p*-values <0.05 considered statistically significant.

## Results

### Baseline characteristics

**Figure 1.**
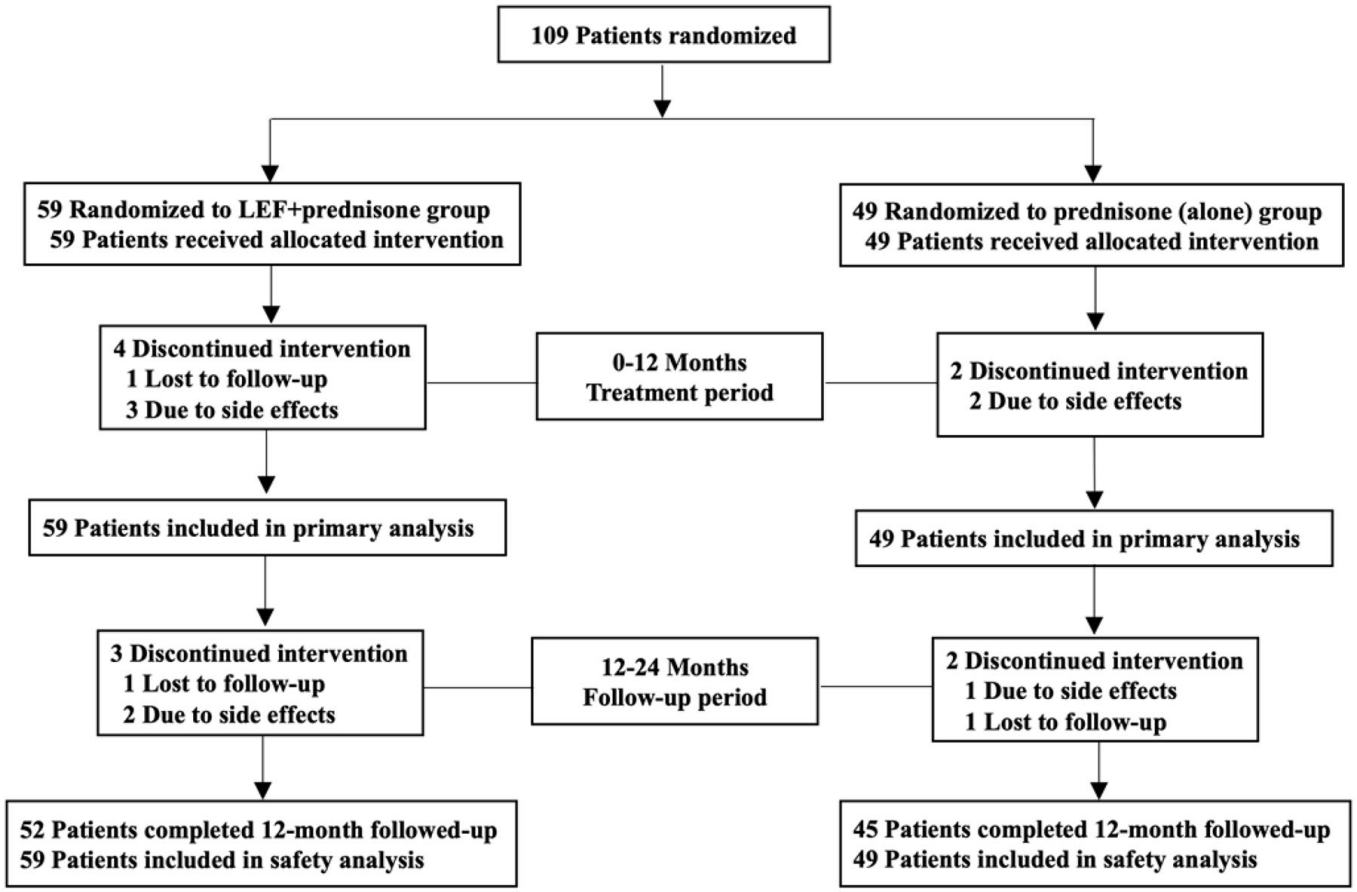
Patient enrollment and follow-up. LEF: leflunomide.

A total of 108 patients were enrolled and eligible for randomization in this study from 1 June 2004 to 30 June 2010 (Figure 1). There were 59 cases in the LEF + prednisone group, including 32 males and 27 females, aged 35.7 ± 11.2 years, and 49 cases in the prednisone (alone) group, including 23 males and 26 females, with an age of 35.5 ± 11.2 years. The baseline characteristics between the two groups were comparable (see Table 1).

**Table 1.** Baseline characteristics.

Characteristics	LEF + prednisone (*n* = 59)	Prednisone (alone) (*n* = 49)	*p*-Value
Male (*n*)	32 (54%)	23 (47%)	0.066
Female (*n*)	27 (46%)	26 (53%)	
Age (years)	35.7 ± 11.2	35.5 ± 11.2	0.185
BMI (kg/m^2^)	22.3 ± 1.3	22.5 ± 1.6	0.886
SBP (mmHg)	123.5 ± 15.6	124 ± 12.9	0.882
DBP (mmHg)	79.3 ± 12.3	79.1 ± 10.1	0.911
Duration of disease (months)	19.4 (4.1–46.8)	10.6 (6.2–40.1)	0.184
UPE (g/24 h)	1.8 (1.3–3.5)	1.9 (1.2–2.9)	0.420
sALB (g/L)	37.7 ± 5.0	37.2 ± 5.1	0.609
BUN (mmol/L)	6.6 ± 2.4	6.0 ± 1.9	0.159
Scr (μmol/L)	99.3 ± 56.8	96.4 ± 38.6	0.199
eGFR (ml/min/1.73 m^2^)	83.1 ± 39.6	84.6 ± 38.5	0.947
Hb (g/L)	132.0 ± 16.6	131.1 ± 19.6	0.797
ALT (IU/L)	20.3 ± 5.8	21.5 ± 11.0	0.470
AST (IU/L)	20.7 ± 11.4	23.6 ± 20.3	0.352
FBG (mmol/L)	5.0 ± 0.4	4.9 ± 0.5	0.492

UPE: urine protein excretion; sALB: Serum albumin; BUN: blood urea nitrogen; Scr: serum creatinine, eGFR: estimated glomerular filtration rate; Hb: hemoglobin; ALT: alanine transaminase; AST: aspartate aminotransferase; LEF: leflunomide; BMI: body mass index; SBP: systolic blood pressure; DBP: diastolic blood pressure; FBG: fasting blood glucose.

*Note*. Values for categorical variables are given as count; values for continuous variables, as mean ± standard deviation or median [IQR]. Normal reference ranges for main lab parameters in our labs are as follows: AST [10–28 U/L], ALT [0–75 U/L], ALB [34–54 g/L], and Scr [45–104 μmol/L].

### Efficacy

Twenty-four hours UPE when patients with IgAN enrolled were 1.8 (1.3–3.5) and 1.9 (1.2–2.9) in LEF + prednisone group and prednisone (alone) group, respectively. After 12 months treatment, 24-h UPE were significantly lower [0.6 (0.3–1.4) *vs.* 1.8 (1.3–3.5), *p* < 0.01] in LEF + prednisone group, [0.6 (0.3–1.0) *vs.* 1.9 (1.2–2.9), *p* < 0.01] in prednisone (alone) group *vs.* baseline value. At months 3, 6, 9 months, 24-h UPE was also significantly lower in both groups compared to baseline data (Table 2). What’s more, the effect sustained during the 12-months follow-up period (Table 2). At 12 months, sALB was significantly higher [44.7 ± 6.4 *vs.* 37.7 ± 5.0, *p* < 0.01] in LEF + prednisone group, [43.3 ± 3.6 *vs.* 37.2 ± 5.1, *p* < 0.01] in prednisone (alone) group *vs.* baseline data. At months 3, 6, 9 months, sALB were also much higher in both groups compared to baseline (Table 2), and the effect was sustained during the 12-months follow-up (Table 2). Scr and eGFR did not change significantly throughout the treatment and follow-up periods (Table 2), indicating renal function was stable in both groups through the treatment period. The difference of the 24-h UPE, sALB, Scr, and eGFR between the two groups was not significant at each visit (*p* > 0.05). It suggested that LEF plus low-dose prednisone and conventionally accepted-dose prednisone had the same effect on the treatment of progressive IgAN.

**Table 2.** Outcomes of treatment.

Characteristics	LEF + prednisone	Prednisone (alone)	*p*-Value
UPE (g/24 h)			
Baseline	1.8 (1.3–3.5)	1.9 (1.2–2.9)	0.318
Month 3	0.8 (0.4–1.9)**	0.9 (0.3–1.3)**	
Month 6	0.8 (0.3–1.9)**	0.8 (0.2–1.6)**	
Month 9	0.6 (0.3–1.8)**	0.7 (0.2–1.0)**	
Month 12	0.6 (0.3–1.4)**	0.6 (0.3–1.0)**	
Month 18	0.4 (0.1–0.9)**	0.5 (0.2–1.1)**	
Month 24	0.5 (0.1–1.1)**	0.5 (0.3–1.0)**	
sALB (g/L)			
Baseline	37.7 ± 5.0	37.2 ± 5.1	0.073
Month 3	40.4 ± 3.8**	40.2 ± 3.4**	
Month 6	41.5 ± 4.6**	42.9 ± 4.4**	
Month 9	43.3 ± 4.2**	42.6 ± 4.0**	
Month 12	44.7 ± 6.4**	43.3 ± 3.6**	
Month 18	44.9 ± 2.8**	42.5 ± 5.2**	
Month 24	44.3 ± 3.0**	43.3 ± 3.8**	
Scr (umol/L)			
Baseline	112.3 ± 56.8	96.4 ± 38.6	0.689
Month 3	107.2 ± 39.4	97.9 ± 36.1	
Month 6	106.5 ± 45.2	94.4 ± 32.4	
Month 9	107.6 ± 36.4	92.3 ± 32.5	
Month 12	111.4 ± 43.4	97.9 ± 42.6	
Month 18	101.3 ± 34.0	98.6 ± 28.4	
Month 24	108.3 ± 26.4	109 ± 65.6	
eGFR (ml/min/1.73 m^2^)			
Baseline	83.9 ± 39.6	84.6 ± 38.5	0.847
Month 3	83.4 ± 26.8	85.4 ± 25.7	
Month 6	84.6 ± 28.6	85.4 ± 28.3	
Month 9	84.2 ± 25.8	85.8 ± 24.6	
Month 12	83.3 ± 25.4	84.8 ± 26.6	
Month 18	83.4 ± 24..8	83.9 ± 25.0	
Month 24	83.2 ± 20.9	83.9 ± 21.5	

UPE: urine protein excretion; sALB: Serum albumin; Scr: serum creatinine, eGFR: estimated glomerular filtration rate; LEF: leflunomide.

**p < 0.01 *vs.* baseline value.

At 12 months, 12 patients had complete remission, 13 patients had partial remission in LEF + prednisone group, and 15 patients had complete remission, 10 patients had partial remission in the prednisone (alone) group. Overall response rates were 69% (36 of 52 patients) in the LEF + prednisone group and 67% (30 of 45 patients) in the prednisone (alone) group. There was no significant difference between the two groups (*p* = 0.959) (Table 3).

**Table 3.** The complete, partial, and overall response between LEF + prednisone group and prednisone (alone) group.

	LEF + prednisone		Prednisone (alone)		*p*-Value
	*n*/*N*	Rate (%)	*n*/*N*	Rate (%)	
Month 12					
CR	17/52	33	18/45	40	0.592
PR	19/52	37	12/45	27	0.411
Overall response	36/52	69	30/45	67	0.959

CR: complete remission; PR: partial remission; n/N: event number/total number.

At 24 months, relapse rates were 3% (two of 59 patients) in the LEF + prednisone group and 10% (five of 49 patients) in the prednisone (alone) group. There was no significant difference between the two groups (*p* = 0.299) (Table 4).

**Table 4.** The relapsing rate between LEF + prednisone group and prednisone (alone) group.

	LEF + prednisone		Prednisone (alone)		*p*-Value
	*n*/*N*	Rate (%)	*n*/*N*	Rate (%)	
Month 24					
Relapsing	2/59	3	5/49	10	0.299

*n*/*N*: event number/total number.

**Figure 2.**
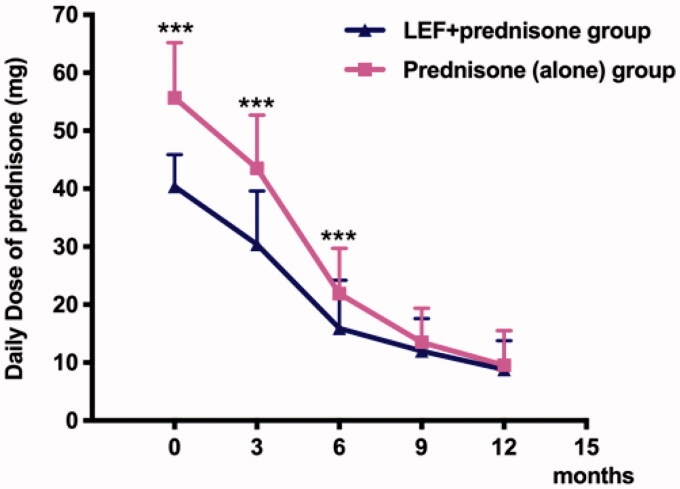
The dosage of prednisone in the LEF + prednisone group was much lower than that in the prednisone (alone) group. ***p < 0.001 *vs.* prednisone (alone) group.

Baseline daily oral prednisone dose in LEF + prednisone group was much lower than that in the prednisone (alone) group (40.4 ± 5.5 *vs.* 55.7 ± 9.5, *p* < 0.001). At 3 and 6 months, daily oral prednisone doses in LEF + prednisone group were 30.4 ± 9.2 and 15.9 ± 8.3, respectively, and they [(30.4 ± 9.2 *vs.* 43.5 ± 9.2, *p* < 0.001); (15.9 ± 8.3 *vs.* 21.9 ± 7.8, *p* < 0.001)] were significantly lower than that in prednisone (alone) group (Table 5, Figure 2). Further, the total amount of prednisone in the LEF + prednisone group was lower than that in the prednisone (alone) group (21.5 ± 13.41 *vs.* 28.83 ± 19.95, *p* = 0.031) (Table 5).

### Adverse events

At the early induction stage, adverse events occurred. Incidence of adverse events and severe adverse events, such as respiratory and pulmonary infection and sepsis were comparable between the two groups (details on adverse event reporting were in Table 6).

**Table 5.** The daily prednisone dose in LEF + prednisone group and prednisone (alone) group.

Treatment time	LEF + prednisone (mg/day)	Prednisone (alone) (mg/day)	*p*-Value
Baseline	40.4 ± 5.5	55.7 ± 9.5	<0.001
Month 3	30.4 ± 9.2	43.5 ± 9.2	<0.001
Month 6	15.9 ± 8.3	21.9 ± 7.8	<0.001
Month 9	12.0 ± 5.6	13.5 ± 5.9	0.181
Month 12	8.8 ± 5.0	9.5 ± 6.0	0.493
Total	21.5 ± 13.4	28.8 ± 20.0	0.031

## Discussion

Patients with IgAN, with overt proteinuria (>1 g/day) and reduced eGFR, are at high risk of progression to ESKD. To our knowledge, this study was the first multicenter RCT study to compare the efficacy and safety of LEF plus prednisone to conventionally accepted-dose prednisone in patients with progressive IgAN. We observed that LEF plus low-dose prednisone is as effective as conventionally accepted-dose prednisone for the treatment of progressive IgAN, with decreased 24-h UPE, increased sALB, stable renal function. Upon completion of the 12-month treatment, after cessation of trial medications, the mean percentage reduction in 24-h UPE was sustained in both groups during the 12-month follow-up period, which was consistent with changes in sALB, Scr, and eGFR. In addition, patients treated with LEF plus low-dose prednisone have comparable overall response rate, relapsing rate, and incidence of adverse events, as compared with that in the conventionally accepted dose prednisone group.

Proteinuria is a known risk factor for the progression of IgAN. Glucocorticoids, an immunosuppressive agent have proved effective in lowering proteinuria. The present study demonstrated that LEF plus low-dose prednisone has a similar effect in the reduction of 24-h UPE *vs.* conventionally accepted-dose prednisone. In addition, the reduction of 24-h UPE sustains throughout the treatment and follow-up periods. Evidence showed that a reduction in proteinuria is associated with a reduced risk of progression to ESKD in patients with IgAN [18]. Our previous study showed that a greater reduction of proteinuria was associated with better outcomes in IgAN patients [13]. In another single-center study, Buardle et al. also confirmed glucocorticoids or combined with immunosuppressive therapy can reduce proteinuria and loss of renal function in patients with progressive IgAN [19]. In addition, Le et al. showed that the rate of decline of renal function was associated with higher levels of time-averaged proteinuria, which was the most important risk factor of progression to renal failure in Chinese adult patients with IgAN [20]. Moreover, a meta-analysis of trials for IgAN suggested that proteinuria reduction was significantly associated with outcomes in ESKD, with each 30% reduction in proteinuria, the risk of ESKD (non-significantly) decreased comparably [21,22]. Therefore, The amount of proteinuria achieved from LEF plus low-dose prednisone was in favor of the outcome of IgAN patients.

Scr and sALB are also associated with the outcome of IgAN patients. At the time of biopsy, eGFR < 60 mL/min per 1.73 m^2^ were the independent risk factors for progression to ESKD in patients with IgAN [20]. We previously reported that the time-averaged serum albumin might serve as a marker of the long-term renal prognosis of IgAN patients who have achieved remission [23]. The present study showed that after treatment with LEF plus low-dose prednisone or conventionally accepted-dose prednisone, sALB significantly increases in both groups, which is in accordance with our previous findings [23]. What’s more, the Scr and eGFR remain stable through the treatment and follow-up periods between the two groups, indicating the renal function is better preserved during the treatment and follow-up periods. The stabilization in Scr and eGFR illustrates that this patient population is at low risk of disease progression, the interventions in this trial for patients with progressive IgAN and overt proteinuria are at least sufficient [24–26], as the remission rate is similar between the LEF + prednisone and prednisone (alone) groups. LEF plus low-dose prednisone treatment might be an alternative method in the treatment of progressive IgAN, with an increase in sALB and stabilization in Scr and eGFR.

A lower dosage of glucocorticoids minimizes adverse effects. As we all know, high-dose systemic glucocorticoids and immunosuppressive treatments cause considerable side effects [27]. An increase of adverse events, such as serious infections, hypertension, osteoporosis, weight gain, and diabetes are associated with high-dose systemic glucocorticoids [8,9]. Elevated liver enzymes, digestive symptoms, and alopecia are related to LEF [11,28]. In the present study, the total amount of initial prednisone was lower in the LEF + prednisone group than that in the prednisone (alone) group (Table 6). Further, the LEF dosage in our study was also less than that in the previously reported LEF monotherapy trials in patients with IgAN [29]. These might be the result of fewer adverse effects. With the above findings, it is suggested that LEF plus low-dose prednisone is probably an alternative option for treatment of progressive IgAN, especially in those patients who were not tolerated high-dose prednisone.

**Table 6.** Adverse events during the treatment period.

	LEF + prednisone (*n* = 59) (*n*, %)	Prednisone (alone) (*n* = 49) (*n*, %)	*p*-Value
Respiratory and pulmonary infection	4 (7)	9 (18)	0.122
Urinary tract infection	0 (0)	1 (2)	0.926
Diarrhea	2 (3)	0 (0)	0.559
Slightly elevated liver enzyme	4 (7)	4 (8)	0.924
Rash	1 (2)	4 (8)	0.257
Elevated blood pressure	1 (2)	1 (2)	0.559
Fever	2 (3)	1 (2)	0.870
Sepsis	0 (0)	1 (2)	0.926
Itching	2 (3)	0 (0)	0.559
Nausea	1 (2)	0 (0)	0.926
Agrypnia	1 (2)	0 (0)	0.926
Paraesthesia	1 (2)	0 (0)	0.926
Insanity	1 (2)	0 (0)	0.926
Lipsotrichia	1 (2)	0 (0)	0.926
Herpes zoster	0 (0)	2 (4)	0.396
Toothache	0 (0)	1 (2)	0.926
Fatigue	0 (0)	1 (2)	0.926
Menstrual disorder	0 (0)	1 (2)	0.926
Insomnia	0 (0)	1 (2)	0.926
Obesity or weight gain	0 (0)	0 (0)	1.000
Impaired glucose tolerance or diabetes	0 (0)	0 (0)	1.000
Cataract	0 (0)	0 (0)	1.000
Acne	0 (0)	0 (0)	1.000
Avascular necrosis of hips	0 (0)	0 (0)	1.000
Total (*n*)	21 (36)	27 (55)	0.066

## Conclusions

In conclusion, this study suggests that LEF plus low-dose prednisone did not differ in reducing proteinuria, increasing sALB, and stabilizing Scr and eGFR, and had comparable adverse events in patients with progressive IgAN. The observed effect was additive to optimized ACEIs or ARBs and supported LEF plus low-dose prednisone might be an effective therapy in patients with IgAN at high risk of progression.

## Limitations

Certain limitations in this trial, with relatively small sample size, short follow-up time. Therefore, the long-term efficacy and safety of the application of LEF plus low-dose prednisone in progressive IgAN patients also need to be quantified in a larger trial of a longer duration. Another limitation of the study is the ethnic difference, thus further studies are required to assess whether the therapeutic benefits exist in non-Asian patients with progressive IgAN. Additionally, this study did not include a LEF-alone arm, we could not compare the efficacy to LEF alone.

## Data Availability

The datasets used and/or analyzed during this study are available from the corresponding author on reasonable request.

## Abbreviations

IgAN: IgA nephropathy
LEF: Leflunomide
UPE: urine protein excretion
sALB: serum albumin
Scr: serum creatinine
eGFR: estimated glomerular filtration rate
ESKD: end-stage kidney failure
ACEIs: angiotensin-converting enzyme inhibitors
ARBs: angiotensin receptor blockers
RAS: renin-angiotensin system
RCT: randomized controlled trial
CR: Complete remission
PR: partial remission
NR: no response
